# The Mediating Role of Social Support in the Relationship Between Parenting Styles and Adolescent Drug Abuse Identification

**DOI:** 10.3389/fpsyg.2021.802408

**Published:** 2022-01-10

**Authors:** Li Liu, Weijie Meng, Bingyuan Liu

**Affiliations:** ^1^School of Educational Science, Ludong University, Yantai, China; ^2^Institute for Education and Treatment of Problematic Youth, Ludong University, Yantai, China; ^3^School of Marxism, Shandong Youth University of Political Science, Jinan, China

**Keywords:** adolescent drug abuse identification, parenting styles, social support, mediating role, prevention and intervention strategies

## Abstract

Adolescent drug abuse is a social issue of global concern, causing a serious burden of diseases for individuals, families and society. To design effective prevention and intervention strategies for adolescent drug abusers, the predictive factors associated with drug abuse must be quantified and assessed. This study explores the similarities and differences between the parenting styles of adolescent drug abusers and non-drug abusers and applies a structural equation model to analyze the mechanisms involved between parenting styles, social support and adolescent drug abuse identification. Data were derived from adolescent drug abusers (*n* = 363) and non-drug abusers (*n* = 229) between the ages of 18 and 35 in China, and the data were collected and analyzed by SPSS 26 and AMOS 24. The results show that parenting styles significantly predict adolescent drug abuse identification, and different parenting styles have different influencing mechanisms, which further indicates that poor parenting styles are a risk factor for adolescent drug abuse. Additionally, social support plays a mediating role between parenting styles and drug abuse identification (χ^2^/df = 4.52, CFI = 0.939, TLI = 0.914, RMSEA = 0.077, IFI = 0.939, PCFI = 0.671). The specific pathways involved are as follows: Paternal parenting style → Social support → Drug abuse identification and Maternal parenting style → Social support → Drug abuse identification. However, beyond this, the mediation model of social support shows good adaptability and stability between adolescent drug abusers and non-drug abusers. Since parenting styles and social support are important predictors of adolescent drug abuse, the importance of integrating family-social support antidrug programs into adolescent prevention and intervention strategies should be considered.

## Introduction

Drug abuse is a social issue of global concern, and an increase in the number of drug abusers has placed a serious burden of disease on individuals, families and society (Kepple, 2017; Perruci et al., 2021). According to statistics, approximately 279 million people worldwide used drugs in 2020, representing a 22% increase from the number of drug users in 2010 (UNODC, 2021). For drug abusers, first exposed to smoke and alcohol occurs between the ages of 12 and 14 (Malta et al., 2011). These findings are worrisome because early exposed to drugs increases the likelihood of physical illness and social risk in adolescents. The findings are also concerning to educators and others who work with adolescents and call for prevention campaigns targeting drug abuse identification (James et al., 2013; Thompson et al., 2013; Hall et al., 2016). Drug abuse identification mainly refers to an individual’s effective or valuable evaluation of drug abuse, emphasizing identification with drug abuse events. Furthermore, drug abuse identification is essentially a value identification of drug abuse events. Therefore, in preventing adolescent drug abuse, it is important to identify and focus on the predictors of protection and risk associated with early exposed to drugs (Degenhardt et al., 2016; Dumas et al., 2020). Additionally, social development theorists believe that the protection and risk factors of adolescent drug abuse are connected to the first socialization model—learning behavior (Cambron et al., 2019). Furthermore, social development theorists believe that family is an influential domain for drug use among adolescents. Adolescents acquire the values, attitudes, cognitive styles and behavioral habits of others based on observation and learning in their households, especially through the continuing influence of parenting styles on the development of their positive and negative behaviors (Karaer and Akdemir, 2019). For instance, as reported by Lippold et al. (2014) and Valente et al. (2019), parenting styles are strong predictors of drug abuse among adolescents.

The classification of parenting styles can be traced back to the work of Baumrind (2013), which is used to explore parents’ responses to adolescents’ attitudes or orientations in various situations or surroundings, mainly in terms of values, attitudes toward parenting styles, development concepts and parenting educational practices for adolescents (Takahara et al., 2017; Clayborne et al., 2021). Moreover, stable parenting styles involve long-term maintenance of values, beliefs, attitudes, and behaviors (Dembo et al., 2015). Parenting styles entail attitudes and behaviors toward adolescents and provide an emotional environment for parent-child relationships and adolescent development. In addition, Schaefer proposed acceptance-rejection, psychological autonomy-psychological control, and firm control-lax control approaches based on children’s evaluation of their own parents’ parenting styles (Matejevic et al., 2014). However, Perris and Andersson (2000) observed that the aforementioned dimensions do not cover all facets of parenting styles. Based on this, the authors constructed a new model and identified four factors that affect parenting styles, including rejection, overprotection, emotional warmth and the favoring subject (Asbrand et al., 2017), which are also the main facets of parenting styles examined in this study.

Parenting styles are also a major focus of adolescent drug abuse identification research. Researchers have found that low levels of support, rejection, and overprotective parenting styles are related to elevated rates of adolescent drug abuse. In contrast, warm understanding and a high level of support can reduce dependence and protect against adolescent drug abuse (Matejevic et al., 2014; Garcia et al., 2020). In addition, the relationship between parenting styles and drug abuse varies across countries depending on culture, but the findings remain relevant in that parenting styles have effects independent of the country investigated (Valente et al., 2017). For instance, some researchers have found that positive parenting styles are protective against adolescent drug abuse in Europe, whereas parental supervision and control are more important than supportive and warm parenting styles in Brazil (Ribeiro et al., 2013; Calafat et al., 2014; Sanchez et al., 2018). Therefore, to improve the prevention and intervention strategies of adolescent drug abusers, the development of parenting styles in accordance with national cultures is crucial.

Despite the evidence for the roles of parenting styles as risk and protective factors, researchers have ignored the mediating effect of parenting styles on adolescent drug abuse identification. Moreover, most studies have tended to examine the relationship between parenting styles and drug abuse identification and have not captured the complexity of the patterns and mechanisms of action involved (Varvil-Weld et al., 2014; Martínez-Loredo et al., 2016; Özdemir and Koutakis, 2016; Reis et al., 2020). Bronfenbrenner identified the family system as an important “microsystem” in which individuals are closely connected that affects the development of individuals and other systems. Such development is also interactive (Guy-Evans, 2020). Moreover, Bronfenbrenner indicated that the matching of microsystems serves as a significant basis for family to play a positive role, especially for a social ecosystem closely linked to the family microsystem (Morse et al., 2014). Social support within the social ecosystem has a strong impact on adolescent drug abuse and is considered to be one of the most important factors in promoting health (Buttram et al., 2013; Holt-Lunstad and Uchino, 2015). Social support includes access to information, material assistance, planning, health advice, and emotional support. Social support serves as a source of information for family, friends, classmates and neighbors, and high-quality social support is of great value in moderating emotions emerging in response to negative life events and in promoting well-being (Rapee et al., 2015; Uchino et al., 2018; Attar-Schwartz et al., 2019). Barra stated that social support, as a general factor contributing to improved stability, is conducive to a more positive perception of one’s surrounding, a better use of personal and social resources, and higher adolescent emotional intelligence (Azpiazu et al., 2015; Gázquez et al., 2016). Barra also indicated that social support is significantly correlated with illicit drug use by adolescents and is a strong predictor of a variable called “Tendency.” Additionally, Birtel et al. (2017) demonstrated that social support received by drug abusers influences illicit drug abuse and relapse after treatment. Furthermore, social support has a positive effect in maintaining the physical, emotional, and mental health of drug abusers, reducing the tendency to use illicit drugs (Mo et al., 2020). In conclusion, parenting styles are an important family-related factor affecting adolescent drug abuse identification, but this relationship lacks process exploration on the possible intermediate link between them, namely, social support. In addition, social support and parenting styles play moderating roles in their relationship with drug abuse (Rudolph et al., 2012; Go et al., 2016; Massah et al., 2017), but scarce studies have assessed the interactions of these moderating effects.

The aim of this study was to explore and analyze the effects of parenting styles and social support on adolescent drug abuse identification. Associated relationships and mechanisms, as well as protective and risk factors involved in drug abuse identification, were investigated. Additionally, differences in the effects of parenting styles on adolescent drug abusers and non-drug abusers were analyzed. Based on this, we propose four hypotheses. First, the parenting styles of parents of drug abusers are more negative than those of adolescent non-drug abusers. Second, parenting styles are significantly correlated with adolescent drug abuse identification. Third, parenting styles influence adolescent drug abuse identification based on the mediating role of social support. Finally, the mediating role of social support has excellent structural stability for both adolescent drug abusers and non-drug abusers. This work provides a practical and theoretical basis for integrating parenting styles and social support into antidrug strategies.

## Materials and Methods

### Participants and Survey Procedures

The purpose of this study was to evaluate the influence of parenting styles and social support on adolescent drug abuse identification. The participants (ages 18–35; *M* = 28.5, *SD* = 3.93) included 363 adolescent drug abusers (49.0% males) from hospitals qualified for drug rehabilitation in Heilongjiang Province and compulsory drug rehabilitation centers in Shandong Province, China, and 229 adolescent non-drug abusers (48.0% males) participating from all walks of life throughout China. Among the adolescent drug abusers, the proportion of first exposed to drugs in different age groups was 14.3% (ages 16), 16.5% (ages 16–18), 47.9% (ages 18–25), and 21.3% (ages 25–35), respectively. The study was completed in 2020.

The participants of this questionnaire agreed to analyze and report on the information they provided in the survey. The procedure was divided into two stages. In the first stage, 363 adolescent drug abusers were evaluated by questionnaire and supervised by relevant staff. Moreover, prior to the completion of the survey, the researchers and relevant staff explained the purpose of the questionnaire and the more difficult questions and offered to answer questions throughout the process. Additionally, during the survey, administered by researchers in the quiet classroom, without the presence of the relevant managers to eliminate any sense of compulsion and the questionnaire was completed within approximately 30–60 min depending on the participants’ personal abilities, then, each de-identified questionnaire was placed in a folder. In the second stage, a random sample of 229 adolescent non-drug abusers (ages 18–35) was selected. The questionnaire followed principles of convenience sampling, was electronic, and presented challenging questions and topic explanations online after questions were answered. Gifts were offered to participants in the study.

### Measures and Instruments

According to the purposes of this study, basic information on population variables (age, sex, age upon first exposure to drugs, etc.) was included as well as the basic measurement indicators for adolescent drug abuse identification, parenting styles, and social support.

#### Drug Abuse Identification

The drug abuse identification scale developed by Weng (2020) was adopted. The scale includes five items designed to represent adolescent drug abuse identification by measuring adolescents’ understanding of drug knowledge, harm from drugs, and drug cognition. The scale was scored on a five-point scale with 1 meaning “never” and 5 meaning “always.” The answers to all questions were summed to obtain an overall score. The higher the score was, the higher the adolescent drug abuse identification level (α = 0.954).

#### Parenting Styles

The EMBU scale described in C. Perris was used to assess parenting styles, including maternal and paternal parenting styles. In the above work, 23 countries were studied for depression, personality disorders, criminal behavior, drug abuse behavior and other psychological behaviors with high reliability and validity (Chen et al., 2020). Based on this study, the revised scale includes 115 items (α = 0.957), among which the subscale for paternal parenting styles has 6 dimensions [with 58 items (α = 0.920)], including emotional warmth and understanding (α = 0.904), punishment and severity (α = 0.916), excessive interference (α = 0.743), favoriting subject (α = 0.794), rejection and denial (α = 0.816), and overprotection (α = 0.708). The maternal parenting style scale has 5 dimensions [with 57 items (α = 0.928)], including emotional warmth and understanding (α = 0.920), punishment and severity (α = 0.903), excessive interference and overprotection (α = 0.798), favoriting subject (α = 0.736), and rejection and denial (α = 0.854). The scale adopts a four-point scoring method where 4 represent “always” and 1 represent “never.” According to the corresponding scoring method, the dimensions of parenting styles were obtained.

#### Social Support

Social support was measured based on the self-rating scale compiled, which includes the three dimensions [with 10 items (α = 0.77)] of subjective support, objective support and support utilization degree (Song and Fan, 2013; Ke et al., 2019). The 4 items of subjective support reflect adolescents’ emotional experiences of and satisfaction with feeling respected, supported and understood. In addition, the 3 items of objective support measure the degree to which adolescents believe that they actually receive support, including direct assistance and social support. The support utilization degree of social support includes 3 items, which reflect the utilization degree of social support by adolescents. The Cronbach’s alpha values of each dimension in this study were 0.65∼0.71.

SPSS 26 was used for data processing, independent sample *t*-test, descriptive statistics, correlation analysis and data mining. AMOS 24 was used to construct a structural equation model to isolate random measurement errors from potential variables and increase explanatory power. Model fit parameters were set by the advocates of the structural equation model and should satisfy: χ^2^/df < 5, SRMR < 0.05, CFI > 0.9, TLI > 0.9, RMSEA < 0.08, IFI > 0.9, and PCFI > 0.5 (Mueller and Hancock, 2019; Abbas et al., 2021). Based on the structural equation model, the bootstrap method was used to analyze the significance of the effect of parenting styles on drug abuse identification.

## Results

### Control and Test of Common Method Deviation

Since all the variables of this study were measured by subject self-reports, anonymous answers and partial item reverse measures were adopted to control common deviation during measurement (Liu et al., 2021). In addition, the Harman single-factor test was applied to the exploratory factor analysis. The unrotated exploratory factor results show 37 factor characteristics with values of greater than 1, and the maximum factor variance explanation rate was measured as 18.796%, which is far less than the critical standard of 40%. Therefore, no serious common method deviation was identified.

### Difference Tests of All Study Variables Between Adolescent Drug Abusers and Non-drug Abusers

An independent sample *t*-test was used to compare the adolescent non-drug abusers and adolescent drug abusers in terms of parenting styles, social support, and drug abuse identification. As shown in Table 1, significant differences were found between the adolescent drug abusers and non-drug abusers in terms of maternal and parental overprotection, social support, and drug abuse identification (*p* < 0.001). Additionally, scores for excessive interferences, favoring subject, rejection and denial, and overprotection from fathers in adolescent drug abusers were significantly higher than those in adolescent non-drug abusers (*p* < 0.001 or *p* < 0.05), whereas the scores for overprotection and excessive interference, rejection and denial, and punishment and severity from mothers were significantly higher in adolescent drug abusers than in the control group (*p* < 0.001 or *p* < 0.05). Furthermore, the results show that the scores for objective support and social support utilization degree in drug abusers were significantly lower than those of adolescent non-drug abusers (*p* < 0.001). The score for drug abuse identification among adolescent drug abusers was significantly higher than that of the adolescent non-drug abusers (*p* < 0.001), confirming that drug abuse identification affects adolescent drug abuse.

**TABLE 1:** Difference tests of all study variables for adolescent drug abusers and non-drug abusers.

Variables		Adolescent drug abusers (*N* = 363) *M* ± *SD*	Adolescent non-drug abusers (*N* = 229) *M* ± *SD*	*t*
Paternal parenting style	Emotional warmth and understanding	39.316 ± 11.659	40.995 ± 11.292	–1.663
	Punishment and severity	18.368 ± 7.328	18.569 ± 7.048	–0.288
	Excessive interference	19.850 ± 5.254	18.768 ± 4.785	2.470*
	Favoriting subject	10.158 ± 2.898	9.536 ± 2.711	2.487*
	Rejection and denial	9.678 ± 3.871	8.901 ± 3.399	2.446*
	Overprotection	12.850 ± 3.419	11.864 ± 3.036	3.485***
Maternal parenting style	Emotional warmth and understanding	39.894 ± 12.418	41.456 ± 11.666	–1.445
	Overprotection and excessive interference	34.855 ± 8.075	31.589 ± 6.367	5.250***
	Rejection and denial	12.904 ± 5.038	11.868 ± 4.127	2.640**
	Punishment and severity	13.266 ± 5.712	12.232 ± 4.152	2.466*
	Favoriting subject	9.183 ± 3.143	9.161 ± 2.251	0.096
Social support	Subjective support	22.253 ± 5.666	22.816 ± 5.883	–1.157
	Objective support	8.132 ± 3.452	10.083 ± 3.95	−6.139***
	Support utilization degree	6.812 ± 2.036	8.009 ± 2.366	−6.319***
Drug abuse identification	Drug abuse identification	15.981 ± 4.753	14.38 ± 5.213	3.843***

Significance levels are indicated by *(for p < 0.05), **(for p < 0.01), and ***(for p < 0.001).

### Correlation Analysis of All Study Variables

Pearson product-moment correlations (Emerson, 2015) were used to calculate the correlation coefficients between parenting styles, social support and drug abuse identification as shown in Table 2. The results show that adolescent drug abuse identification was significantly negatively correlated with social support (*r* = −0.273) and significantly positively correlated with all facets of maternal (*r* = 0.140) and paternal (*r* = 0.145) parenting styles. In addition, we found a significant negative correlation between social support and parenting styles (−0.167 ≤ *r* ≤ −0.162), whereas paternal parenting styles had a significant positive correlation with maternal parenting styles (*r* = 0.285). The results were compared to those of previous studies and show that drug abuse identification was related to maternal and paternal parenting styles and social support. Since many variables were significantly correlated, this laid a foundation for analyzing the effects of parenting styles on adolescent drug abuse identification and the mediating roles of social support.

**TABLE 2:** Correlations, means, and standard deviations of the main study variables.

Variables	*M*	*SD*	Drug abuse identification	Social support	Paternal parenting style	Maternal parenting style
Drug abuse identification	3.072	0.999	1			
Social support	2.612	0.639	−0.273**	1		
Paternal parenting style	1.821	0.499	0.145**	−0.162**	1	
Maternal parenting style	1.731	0.546	0.140**	−0.167**	0.285**	1

*Significance levels are indicated by*

**(for p < 0.01).

### Measurement Model

**FIGURE 1:**
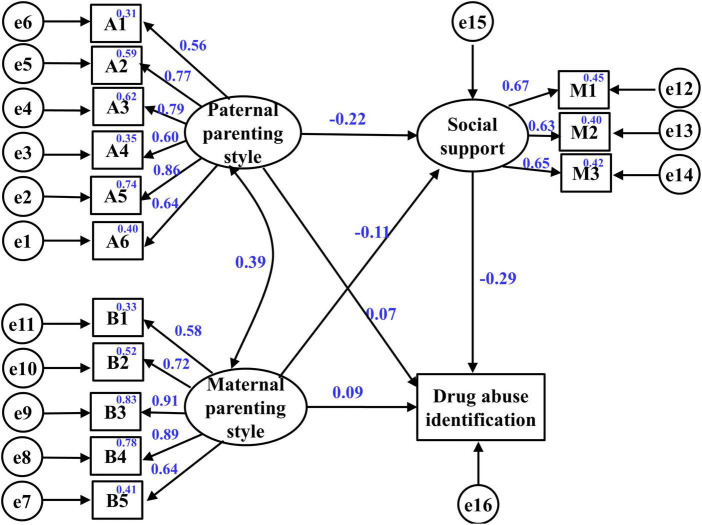
Structural equation model representing the effects of parenting styles, including maternal and paternal parenting styles, and social support on adolescent drug abuse identification.

First, the fitting degree of the structural model must be tested. The three latent variables in the model are assumed to include multiple factors, and each factor involves multiple projects. Thus, to reduce study variable errors, the projects were packaged first, and the project mean of each factor was taken as the new index of each latent variable. Specifically, the mean of each factor for parenting styles, social support, and drug abuse identification, accounting for 15 factors, was used as a new indicator of the latent variables. According to the theoretical basis and research hypothesis of this study, paternal and maternal parenting styles were set as independent variables, social support was set as a mediating variable, and drug abuse identification was set as a dependent variable to construct the structural equation model (Figure 1). Confirmatory factor analysis results show that the measurement model has a good fit: χ^2^/df = 4.520, CFI = 0.939, TLI = 0.914, RMSEA = 0.077, SRMR = 0.046, IFI = 0.939, PCFI = 0.671. However, the standardized factor loads of each index in the model range between 0.555 and 0.914 and above 0.6. Since the load of each factor is greater than 0.4, the structural model shows “goodness of fit” and high structural validity.

### Structural Model: Linking Maternal and Paternal Parenting Styles, Social Support, and Drug Abuse Identification

The measurement model includes the variance and covariance between all latent variables in the model. Therefore, the path coefficient between indicators can be estimated by calculating the variance and covariance of variables, and the recursive form was generally adopted in the selection of models. AMOS was used to calculate the path coefficient as described in Table 3. The results indicate that among the independent variables, maternal (β = 0.200, *p* > 0.05) and paternal (β = 0.129, *p* > 0.05) parenting styles did not directly predict drug abuse identification. The β coefficient of social support to drug abuse identification is -0.629 (*p* < 0.001), indicating that social support had a reverse predictive effect on drug abuse identification. In addition, Table 3 shows that both maternal (β = −0.111, *p* < 0.05) and paternal (β = −0.178, *p* < 0.001) parenting styles had a significant reverse effect on social support.

**TABLE 3:** Path coefficients between maternal and paternal parenting styles, social support, and drug abuse identification.

Paths	Estimate	*SE*	CR	*P*
Paternal parenting style → Social support	–0.178	0.047	–3.769	***
Maternal parenting style → Social support	–0.111	0.038	–1.926	*
Paternal parenting style → Drug abuse identification	0.129	0.085	1.526	0.127
Maternal parenting style → Drug abuse identification	0.200	0.103	1.950	0.051
Social support → Drug abuse identification	–0.629	0.118	–5.318	***

*Significance levels are indicated by*

*(for p < 0.05) and

***(for p < 0.001).

### Bootstrap Test of Path Effect Size of Structural Model

The non-parametric bootstrap method was used to test the significance of two indirect paths (I1 and I2) and two direct paths (D3 and D4) with a self-sampling value of 5,000 as shown in Table 4, which lists two indirect paths in the model: (I1) Paternal parenting style → Social support → Drug abuse identification. The effect value of this path is 0.062 (SE = 0.018, *p* < 0.001, 95% CI [0.032, 0.103]), indicating that under social support, paternal parenting styles can predict adolescent drug abuse behavior. (I2) Maternal parenting style → Social support → Drug abuse identification. The effect value of this path is 0.031 (SE = 0.017, *p* = 0.036 and *p* < 0.05, 95% CI [0.002, 0.069]), demonstrating that maternal parenting styles also predict adolescent drug abuse behavior through social support, which further proves the indirect influence of parenting styles on adolescent drug abuse identification. However, in the direct path (D3 and D4) of parenting styles to drug abuse identification, all of the *p*-values are greater than 0.05, and 95% CI values include 0, indicating that parenting styles have no significant direct predictive effect on drug abuse identification. In conclusion, social support plays a fully mediating role in the influence of maternal and paternal parenting styles on drug abuse identification.

**TABLE 4:** Bootstrap test for significance of the main effect size of the structural model.

Runs	Paths	Standardized effect value	SE	*P*	95% CI
I1	Paternal parenting style → Social support → Drug abuse identification	0.062	0.018	0.000	[0.032, 0.103]
I2	Maternal parenting style → Social support → Drug abuse identification	0.031	0.017	0.036	[0.002, 0.069]
D3	Paternal parenting style → Drug abuse identification	0.072	0.054	0.152	[−0.028, 0.169]
D4	Maternal parenting style → Drug abuse identification	0.089	0.047	0.071	[−0.008, 0.176]

### Multi-Group Analysis of the Mediation Model Considering Social Support as a Mediating Variable

**FIGURE 2:**
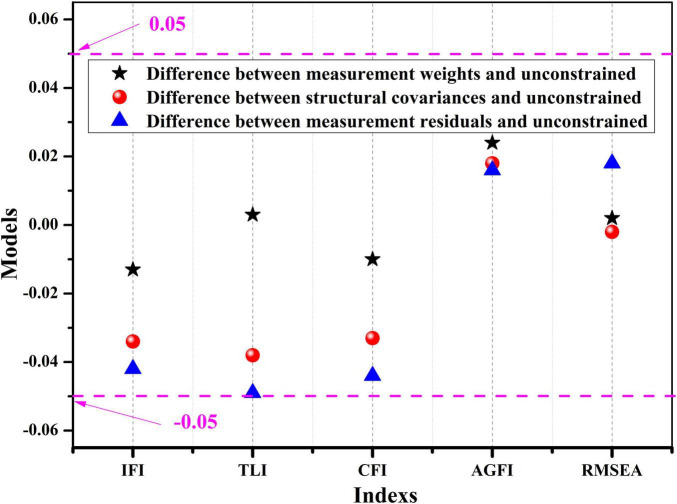
Model invariant tests of adolescent drug abusers and non-abusers (Detla).

Although the aforementioned studies prove the mediating role of social support between parenting styles and drug abuse identification, the mediation model constructed can only be generalized for the overall sample. Since our samples include adolescent drug abusers and non-drug abusers, a multi-group analysis of the mediation model was needed to further verify the model’s suitability for the two types of groups. A confirmatory factor analysis of the multi-group samples is shown in Table 5 and Figure 2. Table 5 shows a good fit between the models of adolescent drug abusers and non-drug abusers (χ^2^/df < 5). Furthermore, the difference values of the fit index factors, including IFI, TLI, GFI, AGFI, and RMSEA, are less than 0.05, indicating no significant differences between the initial model and restricted model (Figure 2); that is, we found no significant differences between adolescent drug abusers and non-drug abusers in this model, and the model exhibited a certain level of stability. In conclusion, the mediation model of adolescent drug abuse is also applicable to adolescent non-drug abusers and can be extended to adolescent non-drug abusers, providing further evidence of the accuracy of the mediation model of social support for adolescent non-drug abusers. Additionally, the structural model provides a theoretical basis and application guidance for adolescent drug abuse prevention and intervention.

**TABLE 5:** Analysis of fit between adolescent drug abusers and non-drug abusers.

Model	χ^2^	χ^2^/df	IFI	TLI	GFI	AGFI	RMSEA
Unconstrained	694.261	4.339	0.885	0.848	0.860	0.790	0.075
Measurement weights	766.907	4.408	0.872	0.845	0.850	0.792	0.076
Structural covariances	837.541	4.679	0.851	0.810	0.827	0.768	0.086
Measurement residuals	891.515	4.819	0.843	0.809	0.816	0.749	0.093

## Discussion

Adolescent drug abuse problems have been widely studied (Tremblay et al., 2020; Trucco, 2020), especially in relation to parenting styles, which have a profound influence on adolescent drug abuse. The relationships and mechanisms between parenting styles and adolescent drug abuse identification in China were investigated. The present study finds a significant positive correlation between poor parenting styles and drug abuse identification. The poorer parenting styles are used, the higher the level of drug abuse identification is, which is consistent with the results of previous studies (Becoña et al., 2012; Irles et al., 2013; Zhang et al., 2020; Zhang and Demant, 2021), whereas the mediating mechanism of the relationship remains largely unknown. This study explored the mechanisms of the relationships between parenting styles, such as maternal and paternal parenting styles, and drug abuse identification based on social support. The present study shows that the influence of parenting styles on adolescent drug abuse identification is realized through the mediating role of social support.

### Effect of Parenting Styles on Adolescent Drug Abuse Identification

The results show that parenting styles, including maternal and paternal parenting styles, could significantly predict adolescent drug abuse behavior (Patte et al., 2017). Different family backgrounds and parenting styles have different effects on adolescent drug abuse, but this further proves that poor parenting styles are a risk factor for adolescent drug abuse (Fernandez-Hermida et al., 2012; Hummel et al., 2013; Zrour et al., 2021). This study concludes that risk factors for parenting styles involved in adolescent development include paternal overprotection, excessive interference, rejection and denial, and favoring subject and maternal punishment and severity, overprotection and excessive interference, and rejection and denial. These relationships may exist because family systems are the safest, healthiest, and most effective environments for the growth and development of adolescents, and parents’ mismatched parenting styles can cause tension in parent-child relationships, family dysfunction, and family environment disharmony. The occurrence of these phenomena may reduce adolescents’ dependence on their families and even weaken or cut off family relationships, creating an imbalance in the family ecosystem and inhibiting the healthy development of adolescents (Šumskas and Zaborskis, 2017; Liu and Visher, 2021). On the other hand, parental warmth and understanding, paternal punishment and severity, and maternal favoritism for participants are likely to be protective factors for adolescent drug abuse prevention (Garcia and Serra, 2019; Lukavska et al., 2020). The negative influence of parenting styles forces adolescent drug abusers to separate from the family environment (Kliewer and Zaharakis, 2014). Adolescent drug abusers will then seek a new environment and change their own development directions and goals. Social resources provide important support for the behavioral development of adolescents who take drugs under these circumstances.

### Mediating Role of Social Support

As a risk factor for drug abuse, poor parenting styles cannot directly affect drug abuse identification but can affect drug abuse identification through the role of social support (Ryan et al., 2010; Tussey et al., 2021). It can be concluded that social support plays a mediating role between parenting styles and drug abuse identification, which means that parenting styles play an indirect role in drug abuse identification. The mechanisms of parenting styles, social support and drug abuse identification are summarized as follows. First, for the family, overprotection and excessive interference from parents, as well as the father favoring the subject, will enhance dependence on the family in the process of socialization, which may lead to a decline in objective support and degree of utilization of social support, as well as enhance drug abuse identification and increasing the likelihood of drug abuse. Second, over the course of a lifetime, rejection and denial from parents and severe punishment by mothers can reduce adolescents’ family dependence (Čablová et al., 2014; Valente et al., 2020). Social support may be the main factor that affects the socialization of adolescents who use drugs, and a lack of social support may affect adolescent drug abuse identification (Fredrick et al., 2018). Insufficient objective support and low degree of social support increase adolescents’ access to drugs and the likelihood of adolescent drug abuse (Becoña et al., 2011; Rubio et al., 2020).

Therefore, social support plays an important role in helping explain and analyze the relationship between parenting styles and drug abuse. This result corroborates Bronfenbrenner’s ecosystem theory. Adolescent drug abuse involves a complex process of interaction between family and social support (Eppler, 2019), mainly because family, as a proximal environment, greatly impacts the direction and effectiveness of adolescent behavioral development (Wolford et al., 2020; Cao et al., 2021). When excessive negative parenting styles manifest, resulting in insufficient family support, adolescents may seek new forms of support, and the focus of socialization may shift from family to peers or other social groups. A lack of social support will lead to the development of new models for adolescents to achieve a new balance (Mitrani et al., 2012; Feeney and Collins, 2015). In an environment with high drug abuse identification, adolescents are more inclined to enter drug abuse groups to integrate into the new environment. Furthermore, under the influence of the group, drug abuse identification is enhanced, which means that a pattern of adolescent drug abuse behavior is more likely to appear than other behaviors. Special attention is given to the negative correlation between social support and drug abuse identification, which provides a basis for the establishment of prevention or intervention systems from enhanced social support.

### Importance and Identification of the Mediating Role of Social Support

This study demonstrates that the structural equation model presents a stable structure and invariability between groups and that parenting styles can predict drug abuse identification through social support. According to the presented comparison of social support for different types of adolescents, the social support of adolescent non-drug abusers (*M* = 40.934, *SD* = 9.318, *P* < 0.001) was found to be stronger than that of adolescent drug abusers (*M* = 37.198, *SD* = 8.411, *P* < 0.001). In particular, we found a significant difference between objective support (*P* < 0.001) and support utilization degree (*P* < 0.001). This indicates that positive social support encourages positive behavior for adolescents, and insufficient social support has a direct negative effect on adolescents’ drug abuse identification.

Additionally, previous studies have focused on directly changing or optimizing parenting styles to change adolescent drug abuse while ignoring the mediating role of social support (Kopak et al., 2012; Szapocznik et al., 2012; Ramadhan et al., 2019). The limitations of previous studies can support new prevention and intervention strategies that can enhance the effectiveness of family education by adjusting social support, especially objective support and the utilization of social support. Families, society and adolescents form a closed ecosystem. When this ecosystem is damaged or unbalanced, adolescent drug abuse identification crises occur (Lian et al., 2016). Our results highlight the importance of adolescent drug abuse prevention and intervention strategies applying the following five strategies. First, antidrug agencies can increase social support, especially through the positive role of subjective support and support utilization degree, to control or restrain the negative factors such as excessive interferences, favoring subject, rejection and denial, and overprotection of fathers, and overprotection and excessive interference, rejection and denial, and punishment and severity of mothers. Meanwhile, antidrug agencies should also optimize and develop positive factors such as emotional warmth and understanding of parents, punishment and severity of fathers and favoriting subject of mothers, so as to control the development of parenting styles of adolescents toward a benign direction. Second, the negative and positive factors of adolescent drug abuse span multiple ecological levels of the family, society and other sectors, and there is an urgent need for multifaceted measures targeted at the family and society. A professional team composed of psychologists, social workers and therapists developed family-social support program based on the mediating role of social support. Through the intervention of social support and parenting style, especially the improvement of parental support, the program aims to jointly resist the negative factors leading to adolescent drug abuse by supervising the study and outdoor risky behavior of adolescents with different sexes, types and stages. Third, schools are the best place to start adolescent drug abuse programs. School-based anti-drug programs need to include family topics, which can enhance parenting skills (communication, rules, discipline, etc.), and communication within the family. Fourth, antidrug agencies should strengthen the development of joint antidrug projects with family and social support. And a diversified prevention system based in a systematic and governance concept must be developed. Finally, families and communities involved in the implementation process should follow laws and regulations, strengthen responsibility provisions and punishment measures, and create a green and drug-free growth environment for adolescents.

### Research Limitations and Prospects

This study has some limitations. First, although the presented model can verify which facets of parenting styles can predict adolescent drug abuse identification, it is mostly applied based on horizontal studies and cannot determine causal relationships. Second, the mediating role of social support can well explain the correlations and mechanisms between parenting styles and drug abuse identification. However, there are many types of social support, leading to different associations with drug abuse identification without further differentiation. Third, in explaining the mediating role of parenting styles in drug abuse identification, social support is accompanied by changes in psychological states; therefore, corresponding physiological indexes can be considered in future studies. Fourth, the sample size is insufficient. In the follow-up study, we will increase the sample size to make the study more convincing and meaningful, and improve the promotion. Fifth, the indexes of structural equation model have certain limitations, and further optimization is needed. For instance, the above-mentioned physiological indexes are included in the study to further optimize the model. Finally, adolescent development emphasizes interactions between the individual and the situation. However, this study examined only the one-way prediction relationships between variables, and the cross-lag model will be used to explore two-way effects in the future.

Despite the shortcomings mentioned above, this study provides a detailed discussion of the relationships between parenting styles, social support, and adolescent drug abuse identification, and the results are relatively reliable, providing a reference for adolescent drug abuse prevention and intervention strategies.

## Conclusion

To summarize, parenting styles, including maternal and paternal parenting styles, can significantly predict adolescent drug abuse behavior. Different parenting styles have different influencing mechanisms, further showing that poor parenting styles are a risk factor for adolescent drug abuse identification. Additionally, parenting styles can indirectly influence adolescent drug abuse identification through social support. The specific pathways involved are as follows: Paternal parenting style → Social support → Drug abuse identification and Maternal parenting style → Social support → Drug abuse identification. Social support plays a fully mediating role. However, the validation of the multi-group model shows that the structural model shows good adaptability and stability between adolescent drug abusers and non-drug abusers. This work provides a practical and theoretical basis for utilizing the mediating role of social support to optimize parenting styles and develop strategies for adolescent drug abuse prevention and intervention.

## Data Availability Statement

The original contributions presented in the study are included in the article/supplementary material, further inquiries can be directed to the corresponding author/s.

## Author Contributions

LL and BL conducted the conceptualization and methodology of the research study. WM was responsible for collecting and analyzing the data. LL completed the writing – original draft and formal analysis. All authors contributed to the article and approved the submitted version.

## Conflict of Interest

The authors declare that the research was conducted in the absence of any commercial or financial relationships that could be construed as a potential conflict of interest.

## Publisher’s Note

All claims expressed in this article are solely those of the authors and do not necessarily represent those of their affiliated organizations, or those of the publisher, the editors and the reviewers. Any product that may be evaluated in this article, or claim that may be made by its manufacturer, is not guaranteed or endorsed by the publisher.
